# Nitroxide Functionalized Antibiotics Are Promising Eradication Agents against Staphylococcus aureus Biofilms

**DOI:** 10.1128/AAC.01685-19

**Published:** 2019-12-20

**Authors:** Anthony D. Verderosa, Rabeb Dhouib, Kathryn E. Fairfull-Smith, Makrina Totsika

**Affiliations:** a School of Chemistry, Physics, and Mechanical Engineering, Queensland University of Technology, Brisbane, Queensland, Australia; b Institute of Health and Biomedical Innovation, Queensland University of Technology, Brisbane, Queensland, Australia; c School of Biomedical Sciences, Queensland University of Technology, Brisbane, Queensland, Australia

**Keywords:** *Staphylococcus aureus*, Calgary Biofilm Device, fluorescent probes, Gram-positive bacteria, antibiofilm, antibiotics, biofilm dispersal, biofilm eradication, fluoroquinolones, infection, nitroxide

## Abstract

Treatment of biofilm-related Staphylococcus aureus infections represents an important medical challenge worldwide, as biofilms, even those involving drug-susceptible S. aureus strains, are highly refractory to conventional antibiotic therapy. Nitroxides were recently shown to induce the dispersal of Gram-negative biofilms *in vitro*, but their action against Gram-positive bacterial biofilms remains unknown. Here, we demonstrate that the biofilm dispersal activity of nitroxides extends to S. aureus, a clinically important Gram-positive pathogen. Coadministration of the nitroxide CTEMPO (4-carboxy-2,2,6,6-tetramethylpiperidin-1-yloxyl) with ciprofloxacin significantly improved the biofilm eradication activity of the antibiotic against S. aureus. Moreover, covalently linking the nitroxide to the antibiotic moiety further reduced the ciprofloxacin minimal biofilm eradication concentration. Microscopy analysis revealed that fluorescent nitroxide-antibiotic hybrids could penetrate S. aureus biofilms and enter cells localized at the surface and base of the biofilm structure. No toxicity to human cells was observed for the nitroxide CTEMPO or the nitroxide-antibiotic hybrids. Taken together, our results show that nitroxides can mediate the dispersal of Gram-positive biofilms and that dual-acting biofilm eradication antibiotics may provide broad-spectrum therapies for the treatment of biofilm-related infections.

## Abstract (precis)

Treatment of biofilm-related Staphylococcus aureus infections represents an important medical challenge worldwide, as biofilms, even those involving drug-susceptible S. aureus strains, are highly refractory to conventional antibiotic therapy. Nitroxides were recently shown to induce the dispersal of Gram-negative biofilms *in vitro*, but their action against Gram-positive bacterial biofilms remains unknown.

## INTRODUCTION

Staphylococcus aureus is a Gram-positive commensal and opportunistic human pathogen that is a major cause of nosocomial and community-acquired infections (1). The inherent ability of S. aureus to attach to medical devices and host tissues and to establish biofilms is a major driver of failing antibiotic therapies and the persistence of chronic infections (2–4). Thus, there is an urgent need for novel strategies for the treatment and eradication of S. aureus biofilms.

Current antimicrobial strategies that are effective against planktonic bacteria often have little or no effect when administered to biofilms (5, 6). During infection, bacteria reside mostly within biofilms but can revert to the planktonic lifestyle by modulating the expression of specific genes (7). Consequently, the development of small molecules with the ability to trigger a cell change from biofilm to the planktonic state has become a promising area of antibiofilm research (8, 9).

One of the most promising small molecules with antibiofilm activity is nitric oxide (NO), which has been shown to inhibit biofilm formation and to trigger biofilm dispersal, in a dose-dependent manner (10–12), in a variety of biofilm-forming bacteria (13). In Pseudomonas aeruginosa, for example, treating a mature biofilm with sublethal NO concentrations (picomolar to nanomolar range) triggers a transition from the sessile (biofilm) state to the motile (planktonic) state, providing a means of effective antibiotic therapy (11, 13). The antibiofilm properties of NO in P. aeruginosa are mediated by regulation of intracellular levels of the secondary messenger bis-(3′-5′)-cyclic dimeric GMP (cyclic di-GMP), which plays a pivotal role in biofilm development; high levels facilitate biofilm formation, while low levels prompt biofilm dispersal (7, 14). However, the targeted and controlled delivery of NO to biological systems as therapy is a challenge due to the reactive nature and short half-life (0.1 to 5 s) (15) of the gaseous molecule. Consequently, methods for circumventing this challenge have been the focus of intensive research over the past several years, with the use of NO donors in biofilm dispersal now being comprehensively documented (16, 17). A variety of NO donor compounds with antibiofilm activity have already been described. However, donor molecules are often themselves inherently unstable (18), necessitating an alternative approach.

Nitroxides are long-lived, stable, free radical species that contain a disubstituted nitrogen atom linked to a univalent oxygen atom (19). Nitroxides are considered sterically hindered, structural mimics of NO because both types of compounds contain an unpaired electron, which is delocalized over the nitrogen-oxygen bond. The biological activity of nitroxides is often attributed to their NO-mimetic properties, with both types of compounds being efficient scavengers of protein-derived radicals (20). Nitroxides are mostly air-stable crystalline solids, however, in contrast to NO, which is unstable and gaseous at room temperature. This significant difference makes nitroxides ideal candidates for circumventing the handling and delivery issues associated with NO.

**FIG 1:**
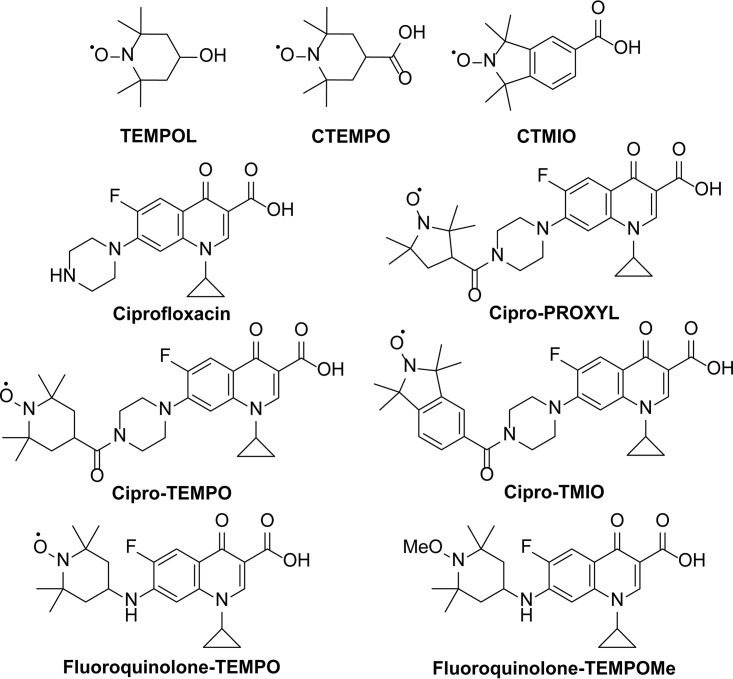
Chemical structures of TEMPOL, CTEMPO, CTMIO, ciprofloxacin, cipro-PROXYL (25), cipro-TEMPO (25), cipro-TMIO (25), fluoroquinolone-TEMPO (26), and fluoroquinolone-TEMPOMe (26).

We previously demonstrated the ability of nitroxides to inhibit and to disperse bacterial biofilms of P. aeruginosa and Escherichia coli (21, 22). Nitroxides were also shown to enhance biofilm antibiotic susceptibility when coadministered as a combined treatment (23). Furthermore, we showed that, by covalently tethering a nitroxide to the antibiotic ciprofloxacin (i.e., cipro-PROXYL [1-cyclopropyl-6-fluoro-7-(4-(2,2,5,5-tetramethyl-1-oxy-pyrrolidine-3-carbonyl)piperazin-1-yl)-4-oxo-1,4-dihydroquinoline-3-carboxylic acid], cipro-TEMPO [1-cyclopropyl-6-fluoro-7-(4-(2,2,6,6-tetramethyl-1-oxy-piperidine-4-carbonyl)piperazin-1-yl)-4-oxo-1,4-dihydroquinoline-3-carboxylic acid], and cipro-TMIO [1-cyclopropyl-6-fluoro-7-(4-(1,1,3,3-tetramethylisoindolin-2-yloxyl-5-carbonyl)piperazin-1-yl)-4-oxo-1,4-dihydroquinoline-3-carboxylic acid]) (Fig. 1) and thus delivering the antibiotic at the site of nitroxide-mediated dispersal, we could achieve effective eradication of mature P. aeruginosa biofilms (24, 25). Furthermore, we also recently developed several profluorescent fluoroquinolone-nitroxide probes (switch-on fluorescent probes) (e.g., fluoroquinolone-TEMPO [1-cyclopropyl-6-fluoro-7-((2,2,6,6-tetramethyl-1-oxy-piperidine-4-yl)amino)-4-oxo-1,4-dihydroquinoline-3-carboxylic acid]) (Fig. 1) for investigation of the interactions of nitroxide functionalized antibiotics with bacterial cells (26). While the dispersal and antibiofilm properties of nitroxides against Gram-negative bacteria have been successfully documented, their antibiofilm properties against Gram-positive bacteria remain unexplored. Here, we employed a reproducible, high-throughput, *in vitro* biofilm assay to comprehensively examine the full antimicrobial, antibiofilm, and biofilm eradication potential of nitroxides and antibiotic-nitroxide hybrids against S. aureus. Our findings demonstrate the antibiofilm action of nitroxides against a clinically important Gram-positive pathogen, extending this promising therapeutic strategy to a large number of S. aureus biofilm-related infections.

## RESULTS

### The nitroxide CTEMPO can disperse established S. aureus biofilms.

**FIG 2:**
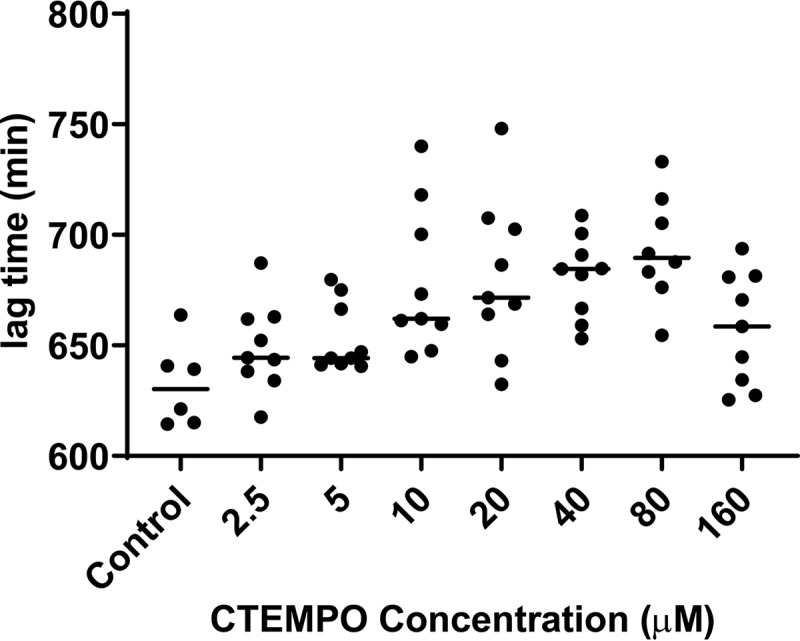
Nitroxide-mediated S. aureus ATCC 29213 biofilm dispersal. Established S. aureus biofilms were treated for 24 h with the nitroxide CTEMPO (at a concentration of 160 to 2.5 μM) and ciprofloxacin (6 μM) or with ciprofloxacin alone (6 μM) (control). Biofilm-associated bacteria were recovered after treatment and enumerated as described in Materials and Methods. The lag times from growth curves for recovered bacteria were calculated using nonlinear regression. Dot plots show data from treated and control biofilms obtained from six biological replicates. Lines show group medians. Group medians were compared using the Kruskal-Wallis test (*P* = 0.0003).

Established S. aureus ATCC 29213 biofilms were treated for 24 h with CTEMPO (4-carboxy-2,2,6,6-tetramethylpiperidin-1-yloxyl) (160 to 2.5 μM) and ciprofloxacin (6 μM) or with ciprofloxacin alone (6 μM). Viable bacteria remaining in the treated biofilms were recovered in medium without nitroxide or antibiotics and monitored for growth, with the lag time recorded as a direct measure of the initial CFU present in each well. Lag time was found to be inversely proportional to the number of bacterial cells in the biofilm that survived antimicrobial treatment (short lag times indicate that many cells survived antimicrobial treatment, while longer lag times indicate that fewer cells survived antimicrobial treatment). Significant increases in lag time, indicative of significant reductions in biofilm-associated bacteria recovered posttreatment, were observed at CTEMPO concentrations in the range of 10 to 80 μM (*P* = 0.0003, Kruskal-Wallis test), compared to control biofilms treated with ciprofloxacin alone (Fig. 2). Because no antimicrobial activity against planktonic or biofilm-residing S. aureus ATCC 29213 cells was observed for CTEMPO alone at those concentrations (Table 1), the nitroxide was most likely inducing cell dispersal in biofilms, not bacterial killing. CTEMPO alone (no ciprofloxacin) had no impact on the number of viable bacteria recovered from the biofilm (data not shown).

**TABLE 1:** MIC and MBEC values for ciprofloxacin, CTEMPO, CTMIO, and various cotreatments and antibiotic-nitroxide hybrids against S. aureus ATCC 29213

Treatment	MIC (μM)^*a*^	MBEC (μM)^*b*^
Control treatment		
Ciprofloxacin	1.5	4,096
CTEMPO	>3,200^*c*^	>1,200^*c*^
CTMIO	>2,048^*c*^	>2,048^*c*^
Cotreatment		
Ciprofloxacin and CTEMPO (2.5 μM)	ND	4,096
Ciprofloxacin and CTEMPO (8 μM)	ND	256
Ciprofloxacin and CTEMPO (10 μM)	ND	512
Ciprofloxacin and CTEMPO (20 μM)	ND	512
Ciprofloxacin and CTEMPO (40 μM)	ND	512
Ciprofloxacin and CTEMPO (80 μM)	ND	2,048
Ciprofloxacin and CTMIO (8 μM)	ND	1,024
Hybrid treatment		
Cipro-PROXYL	6.3	1,024
Cipro-TEMPO	12.5	>1,024^*c*^
Cipro-TMIO	1.5	64
Fluoroquinolone-TEMPO	20	720
Fluoroquinolone-TEMPOMe	>1,200^*c*^	>1,480^*c*^

a MICs were determined via the broth microdilution method, in accordance with CLSI guidelines (49). ND, not done.

b MBECs were determined using the CBD.

c Highest concentration tested.

### Nitroxide coadministration improves the efficacy of ciprofloxacin against S. aureus biofilms.

Because nitroxides appear to induce S. aureus biofilm dispersal, we sought to examine whether this could improve the efficacy of ciprofloxacin against established S. aureus biofilms. Ciprofloxacin is a commonly prescribed fluoroquinolone antibiotic with potent antimicrobial activity against planktonic S. aureus ATCC 29213 (27). We confirmed the ciprofloxacin MIC for this strain to be within the previously published range (1.5 to 0.4 μM) (Table 1) (27). Despite the low MIC, established S. aureus ATCC 29213 biofilms required 4,096 μM ciprofloxacin for complete (99.9%) eradication (concentration range tested, 4,096 to 32 μM), demonstrating the biofilm’s extremely high antibiotic tolerance. The minimum biofilm eradication concentration (MBEC) for ciprofloxacin against established S. aureus ATCC 29213 biofilms was thus determined to be 4,096 μg/ml (Table 1).

Coadministration of CTEMPO (at 2.5, 8, 10, 20, 40, or 80 μM) with ciprofloxacin (at 4,096 to 32 μM) against established S. aureus ATCC 29213 biofilms resulted in significant reductions in the MBEC value for ciprofloxacin at CTEMPO concentrations between 8 and 80 μM (Table 1). Ciprofloxacin potentiation was greatest with 8 μM CTEMPO coadministration, which reduced the ciprofloxacin MBEC value from 4,096 μM to 256 μM, representing a 16-fold improvement in the drug’s efficacy against Gram-positive S. aureus ATCC 29213 biofilms (Table 1). To confirm the synergistic effects of nitroxides and ciprofloxacin against S. aureus biofilms, we performed cotreatment with a different nitroxide, CTMIO (5-carboxy-1,1,3,3-tetramethylisoindolin-2-yloxyl). CTMIO was also able to potentiate the action of ciprofloxacin against established S. aureus ATCC 29213 biofilms (4-fold improvement), without possessing antimicrobial activity alone against planktonic or biofilm-residing cells (Table 1). Taken together, these results demonstrate the ability of nitroxides to significantly improve the efficacy of antibiotic therapy against S. aureus biofilms.

### Ciprofloxacin-nitroxide hybrids are potent S. aureus biofilm eradication agents.

Considering the synergistic effects of ciprofloxacin-nitroxide coadministration on S. aureus biofilm eradication, we evaluated whether combining the nitroxide and antibiotic within one molecule would offer greater improvement. We rationalized that, by localizing the antibiotic directly at the site of nitroxide-mediated dispersal, it could eradicate dispersed cells more effectively than in combination treatment. To test this hypothesis, we utilized several ciprofloxacin-nitroxide hybrids that we generated previously (Fig. 1) (25, 26). Cipro-PROXYL, cipro-TEMPO, and cipro-TMIO (Fig. 1) were first screened in MIC assays for activity against planktonic S. aureus ATCC 29213 (Table 1). All compounds were determined to possess potent S. aureus ATCC 29213 activity (MIC range of 1.5 to 12.5 μM), with cipro-TMIO being the most active (MIC of 1.5 μM, the same as the ciprofloxacin MIC). Subsequent minimum bactericidal concentration (MBC) analysis of cipro-TMIO and ciprofloxacin revealed that cipro-TMIO was at least twice as bactericidal as ciprofloxacin against S. aureus ATCC 29213 cells (cipro-TMIO MBC, 1.5 μM; ciprofloxacin MBC, 3.0). Then, established S. aureus ATCC 29213 biofilms were treated with cipro-PROXYL, cipro-TEMPO, cipro-TMIO, fluoroquinolone-TEMPO, or fluoroquinolone-TEMPOMe (1-cyclopropyl-6-fluoro-7-((1-methoxy-2,2,6,6-tetramethylpiperidine-4-yl)amino)-4-oxo-1,4-dihydroquinoline-3-carboxylic acid) (a fluoroquinolone-;TEMPO derivative with the free radical removed). Cipro-PROXYL, cipro-TMIO, and fluoroquinolone-TEMPO all exhibited potent biofilm eradication activity (Table 1). However, cipro-TMIO was by far the most potent agent, with an MBEC value of 64 μM; this made cipro-TMIO ≥16-fold more potent than ciprofloxacin-CTMIO cotreatment (MBEC, 1,024 μM) and ≥64-fold more potent than ciprofloxacin alone (MBEC, 4,096 μM) against S. aureus biofilms. These results demonstrate the advantage of covalently linking a nitroxide moiety to an antibiotic to enhance its activity against Gram-positive biofilms. Furthermore, ciprofloxacin-nitroxide hybrids were found to be stable in air at room temperature over a 12-month period, with no loss of activity in MIC assays (data not shown).

### Fluoroquinolone-nitroxide hybrids can penetrate S. aureus biofilms and enter surface- and base-residing cells.

To investigate the enhanced activity of nitroxide-antibiotic hybrids against established S. aureus biofilms at the cellular level, we utilized fluoroquinolone-TEMPO, a previously reported profluorescent fluoroquinolone-nitroxide that exploits the inherent fluorescence of fluoroquinolones and the fluorescence-quenching properties of the nitroxide free radical (26). We showed previously that fluoroquinolone-CTEMPO was active against S. aureus planktonic cells and acted as a switch-on fluorescent probe, emitting bright fluorescence upon cell entry (26). These properties make fluoroquinolone-TEMPO a valuable tool for visualizing S. aureus biofilm eradication by nitroxide-antibiotic hybrids. We first established the MBEC value for fluoroquinolone-TEMPO (MBEC, 720 μM) (Table 1) and found fluoroquinolone-TEMPO to be ≥5 times more potent than ciprofloxacin for treatment of S. aureus ATCC 29213 biofilms, further supporting the increased potency of tethered nitroxide-antibiotic hybrids. Importantly, we showed that this potentiation effect was specific to the presence of the free radical nitroxide, as the methoxyamine derivative fluoroquinolone-TEMPOMe, which lacks the nitroxide free radical, lost its antibacterial activity (MIC, >1,200 μM; MBEC, >1,480 μM) (Table 1).

**FIG 3:**
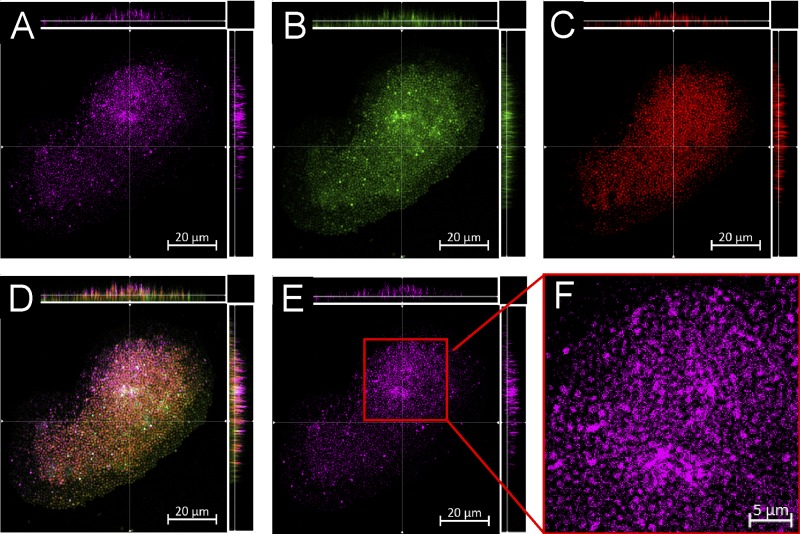
CLSM images of established S. aureus ATCC 29213 biofilms treated for 2 h with fluoroquinolone-TEMPO (150 μM, a sublethal concentration) and then stained with SYTO9 and PI (see Materials and Methods). (A and E) Excited with a 720-nm multiphoton laser (fluoroquinolone-TEMPO [pink]). (B) Excited with a 488-nm laser (SYTO9, live cells [green]). (C) Excited with a 561-nm laser (PI, dead cells [red]). (D) Overlay of images A to C. The light gray cells result from merging of green and pink. (F) Expanded view of image E. Scale bars, 20 μm (A to E) or 5 μm (F). Micrographs show representative horizontal (*x-y*) sections collected within each biofilm, with images A to E also showing vertical sections representing the *y-z* (right) and *x-z* (top) planes, taken at the positions indicated by the lines.

**FIG 4:**
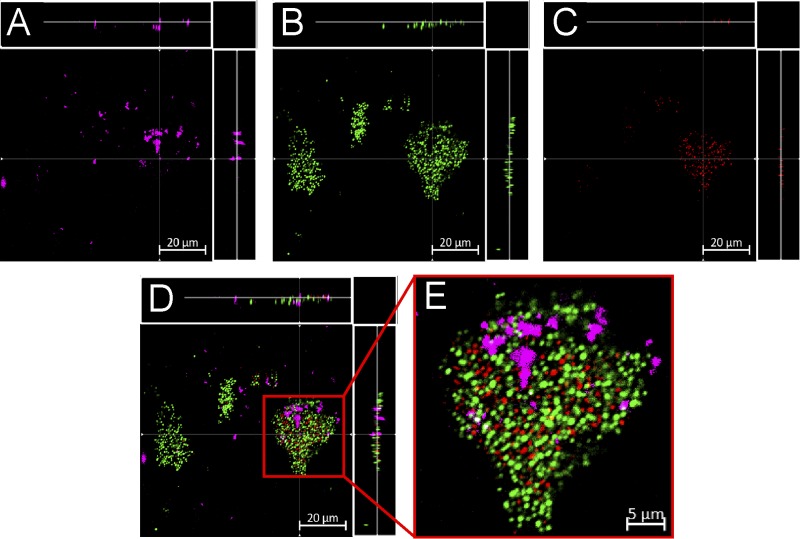
CLSM images of established S. aureus ATCC 29213 biofilms treated for 2 h with fluoroquinolone-TEMPOMe (150 μM) and then stained with SYTO9 and PI (see Materials and Methods). (A) Excited with a 720-nm multiphoton laser (fluoroquinolone-TEMPOMe [pink]). (B) Excited with a 488-nm laser (SYTO9, live [green]). (C) Excited with a 561-nm laser (PI, dead [red]). (D) Overlay of images A to C. (E) Expanded view of image D. Scale bars, 20 μm (A to D) or 5 μm (E). Images A to D show the *x-y*, *y-z*, and *x-z* dimensions.

We then treated established S. aureus biofilms with sub-MBEC concentrations of fluoroquinolone-TEMPO or the fluoroquinolone-TEMPOMe control, stained the treated biofilms for live/dead cells, and examined them using confocal laser scanning microscopy (CLSM). Fluoroquinolone-TEMPO, which acts as a switch-on fluorescent probe, becoming fluorescent upon cell entry, was found to penetrate the S. aureus ATCC 29213 biofilms, entering both surface- and base-residing cells (Fig. 3A, horizontal and vertical side panels). Upon cell entry, the fluorescence of fluoroquinolone-TEMPO was no longer quenched by the free radical nitroxide, and its presence was clearly visible inside biofilm-residing cells (Fig. 3F). Intriguingly, fluoroquinolone-TEMPOMe, which is constitutively fluorescent due to its lack of the free radical nitroxide (conversion to methoxyamine), did not appear to enter biofilm-residing cells; instead, it appeared to be confined to the intercellular space of the biofilm, where extracellular polymeric substances (EPS) are typically found (Fig. 4E). Taken together, these results suggest that the nitroxide free radical likely facilitates the entry of the antibiotic into cells, explaining the enhanced potency of the antibiotic against S. aureus biofilms.

### Fluoroquinolone-TEMPO and CTEMPO are nontoxic to human cells.

Our findings suggest that antibiotic-nitroxide hybrids such as fluoroquinolone-TEMPO have potential in future clinical applications. As part of their early-stage preclinical evaluation, the general toxicity of fluoroquinolone-TEMPO and CTEMPO to human epithelial cells was investigated. No cytotoxicity against human epithelial T24 cells was observed in lactate dehydrogenase (LDH) assays following 24-h cell exposure to fluoroquinolone-TEMPO or CTEMPO at concentrations ranging from 20 to 720 μM (IC_50_, >720 μM).

## DISCUSSION

Currently, treatment options for S. aureus biofilm-related infections are limited, with conventional antibiotics often exhibiting little to no therapeutic effect against biofilm-residing bacteria (5, 6). Consequently, treatment for biofilm infections usually involves prolonged high doses of antibiotics and/or surgical removal of infected tissue or implanted medical devices (28). Accordingly, methods for improving the biofilm eradication activity of conventional antibiotics are urgently needed. Recent studies have demonstrated that nitroxides can mediate biofilm dispersal, which can increase the efficacy of the commonly prescribed antibiotic ciprofloxacin against bacterial biofilms (23–25). Until now, however, the antibiofilm properties of nitroxides have been demonstrated only against two Gram-negative pathogens, P. aeruginosa and E. coli (21–25). In this study, we showed that biofilms of the clinically important Gram-positive pathogen S. aureus are also susceptible to the biofilm dispersal properties of nitroxides.

Our findings demonstrate that nitroxide-mediated biofilm dispersal of the Gram-positive pathogen S. aureus occurs over a micromolar concentration range (5 to 80 μM), which is similar to our previous findings for the Gram-negative pathogens P. aeruginosa and E. coli (20 μM) (21, 23). While the precise mechanism of biofilm dispersal by nitroxides is currently unknown, nitroxides are considered NO mimics and thus their biofilm-dispersing action potentially involves inhibition of intracellular levels of cyclic di-GMP, which has been directly linked to NO-mediated biofilm dispersal in several Gram-negative and Gram-positive bacterial species (14, 29, 30). The signaling nucleotide cyclic di-GMP has been shown to play a pivotal role in biofilm formation and control, with higher levels facilitating biofilm formation and lower levels triggering biofilm dispersal (31–33). Interestingly, our finding that the optimal nitroxide concentrations for biofilm dispersal are similar for Gram-positive and Gram-negative species may suggest that the mechanisms by which nitroxide-mediated dispersal occurs are similar between these species.

Our study demonstrates that the nitroxide CTEMPO potentiates the activity of ciprofloxacin against S. aureus biofilms, significantly improving its eradication efficacy (≥16-fold MBEC improvement). This is consistent with our previous results using nitroxide-ciprofloxacin combination treatment of established P. aeruginosa and E. coli biofilms (23). Interestingly, studies that utilized NO (instead of nitroxides) in combination with antimicrobials reported similar improvements in antimicrobial activity (e.g., a 20-fold increase in the activity of chlorine against multispecies biofilms) (13, 17), supporting similarity between the mechanisms of NO- and nitroxide-mediated biofilm dispersal. Importantly, the ability of nitroxides to enhance the biofilm eradication activity of antimicrobials against biofilms is comparable to that of other leading methods, such as the use of d-amino acids (8-fold improvement over antibiotic alone) (34) and *cis*-2-decenoic acid (≥4-fold improvement over antibiotic alone) (35). Taken together, these results support the use of nitroxides as effective enhancers of antibiotic biofilm eradication activity.

As an improvement over cotreatment, a currently emerging alternative has been the development of dual-acting hybrid compounds. Such compounds combine the antibiofilm activity of a dispersal agent with an antimicrobial agent to produce a hybrid compound that can both disperse and eradicate biofilm-residing cells. We previously utilized this strategy to produce ciprofloxacin-nitroxide hybrids that exhibited potent P. aeruginosa and E. coli biofilm eradication activity (24, 25, 36). In this study, we showed that ciprofloxacin-nitroxide hybrids are also potent S. aureus biofilm eradication agents. Importantly, cipro-TMIO was able to completely eradicate (99.9%) established S. aureus biofilms at a concentration of only 64 μM (MBEC, 64 μM), which is very similar to its P. aeruginosa biofilm eradication activity (94% eradication at 20 μM) (25). Furthermore, the biofilm eradication activity of cipro-TMIO is comparable to that of other promising compounds currently being developed as S. aureus biofilm eradication agents, such as halogenated phenazines (MBEC, 10 μM) (37) and quaternary ammonium compounds (MBEC, 25 μM) (38).

Interestingly, in the case of cipro-TMIO, the addition of the nitroxide moiety to the ciprofloxacin core structure did not negatively affect the activity of the hybrid against planktonic S. aureus cells (MIC of 1.5 μM, the same as the ciprofloxacin value) and even appeared to improve its bactericidal activity (MBC of 1.5 μM, a 2-fold improvement over the ciprofloxacin value). This trend was not evident in our previous study, in which cipro-TMIO was administered to P. aeruginosa planktonic cells (MIC of 160 μM, a ≥100-fold increase over the ciprofloxacin value) (25). These results suggest that the presence of the nitroxide at the secondary amine of ciprofloxacin does not interfere with and may even facilitate the fluoroquinolone’s mode of action (inhibition of DNA gyrase and/or topoisomerase IV) in S. aureus cells. Thus, it appears that ciprofloxacin-nitroxide hybrids produced via functionalization at the secondary amine of ciprofloxacin may be more suitable for the treatment of S. aureus infections, compared with P. aeruginosa infections. Also, the activity of cipro-TMIO against both planktonic and biofilm-residing S. aureus cells and its lack of toxicity to human cells (25) make it an ideal candidate for the treatment of a large number of S. aureus-related infections.

While no general toxicity to human cells was observed for CTEMPO or any of the nitroxide functionalized antibiotics tested to date, future animal studies could explore specific physiological effects linked to nitroxide administration. In particular, some nitroxides have been shown to exhibit vasodilatory properties (39), and their potential use as antihypertensive drugs has been extensively explored (40–45). One of the most potent nitroxide-based vasodilators, and hence the most promising antihypertensive drug candidate, is TEMPOL (4-hydroxy-2,2,6,6-tetramethylpiperidine-1-oxyl) (Fig. 1). This nitroxide, which is structurally similar to CTEMPO (a piperidine-based nitroxide) is currently the lead antihypertensive candidate, having shown efficacy in several animal-based studies (40–45). Interestingly, it appears that the vasodilatory properties of TEMPOL may not extend to other tetra-substituted nitroxides, specifically non-piperidine-based nitroxides such as 3-carbamoyl-2,2,5,5-tetramethylpyrrolidin-1-yloxy (a pyrrolidine-based nitroxide), a result that suggests that the six-membered heterocyclic ring of TEMPOL may be necessary for the vasodilatory properties of this nitroxide (39). In addition, the vasodilatory properties of TEMPOL were shown to be highly concentration dependent, with the minimum dose required to elicit a vasodilation response in a pig model being 145 μM (39). However, effective antihypertensive activity required much higher doses (≥1 mM) (40–42). Considering that, in our study, (i) only 8 μM CTEMPO could effectively potentiate the action of ciprofloxacin against S. aureus biofilms, (ii) cipro-TMIO is not a piperidine-based nitroxide, and (iii) cipro-TMIO has an active concentration of 64 μM, it is reasonable to assume that our compounds would be unlikely to induce vasodilation at concentrations used for the treatment of biofilm-related infections. Nevertheless, their vasodilatory/antihypertensive properties would be worth exploring in the future.

This study has demonstrated that both cotreatment (nitroxide with ciprofloxacin) and ciprofloxacin-nitroxide hybrid (cipro-TMIO) treatment exhibit potent planktonic and biofilm eradication activity against S. aureus ATCC 29213 (methicillin-susceptible S. aureus). However, ciprofloxacin and other fluoroquinolones are no longer effective against resistant S. aureus (46). Instead, other antibiotics, such as vancomycin and rifampin, have become the preferred treatments for methicillin-resistant S. aureus infections (46, 47). While this study has focused on restoring the activity of the fluoroquinolone class of antibiotics, we are hopeful that a similar approach might also be effective for other antibiotic classes.

The profluorescent nitroxide fluoroquinolone-TEMPO is a biofilm eradication agent with the same antibiofilm mechanism as cipro-TMIO. Thus, by utilizing the probe properties of fluoroquinolone-TEMPO, we demonstrated that the state of the nitroxide (free radical) remains unchanged while it interacts with the EPS of the biofilm (i.e., the fluorescence of fluoroquinolone-TEMPO is not switched on in the EPS or prior to cell entry). However, as fluoroquinolone-TEMPO enters the intracellular space, its fluorescence is quickly restored, indicating that the free radical nitroxide has undergone a chemical change. Furthermore, because our results indicate that the presence of the free radical nitroxide is fundamental to the biofilm eradication activity of fluoroquinolone-TEMPO, it can be inferred that (i) the free radical must be involved in the antibiofilm activity of nitroxides and (ii) the antibiofilm role of nitroxides must occur via interference and/or regulation of an intracellular process. These findings support the hypothesis that nitroxides like NO likely regulate the intracellular levels of cyclic di-GMP.

**FIG 5:**
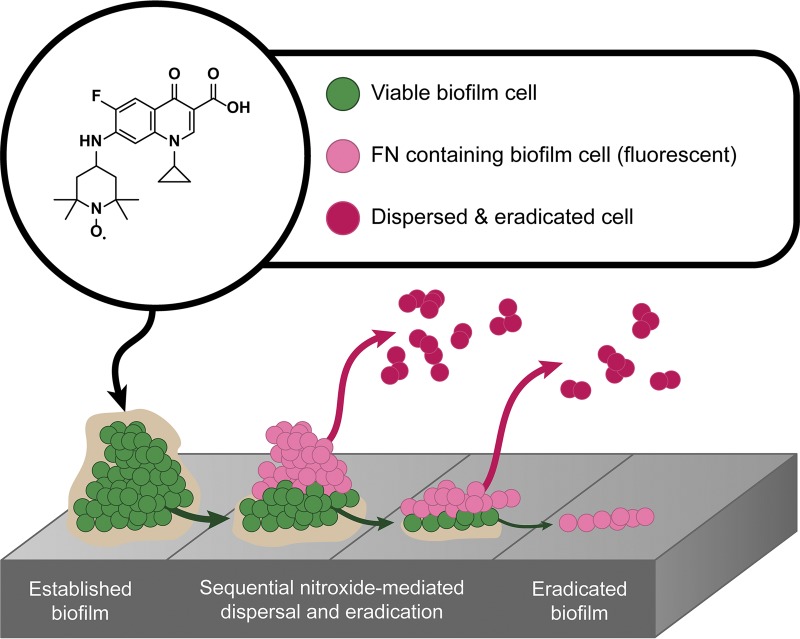
Working model for the dual-action mechanism of fluoroquinolone-TEMPO against S. aureus biofilms. Upon administration, the functionalized antibiotic fluoroquinolone-TEMPO enters surface-residing biofilm cells and induces their dispersal from the biofilm in a concentration-specific manner. Dispersed cells are no longer tolerant to the antibiotic and are thus eradicated, exposing the next layer of biofilm-residing cells to the hybrid compound. Fluoroquinolone-TEMPO enters the newly exposed cells, and the process is repeated until the biofilm is completely eradicated. FN, fluoroquinolone-nitroxide.

Hence, the results presented here support the hypothesis that, much like cotreatment (nitroxide and antibiotic), the biofilm eradication activity of nitroxide functionalized antibiotics, such as cipro-TMIO and fluoroquinolone-TEMPO, may occur via a dual-action mechanism, in which the nitroxide moiety triggers biofilm dispersal and the antibiotic moiety subsequently eradicates the dispersed cells, which are no longer antibiotic tolerant (Fig. 5). However, this alone does not appear to explain the additional efficacy that nitroxide functionalized antibiotics exhibit, compared with cotreatment. Interestingly, our results also indicate that fluoroquinolone-TEMPO can penetrate S. aureus biofilms and enter both surface- and base-residing cells. It is conceivable that this additional penetration and the dual actions (dispersal and eradication) both contribute to the biofilm eradication activity of cipro-TMIO.

In conclusion, we have demonstrated that the biofilm dispersal activity of nitroxides is not limited to Gram-negative pathogens but extends to important Gram-positive pathogens. This work also highlights the ability of nitroxides to restore the antibacterial activity of ciprofloxacin against S. aureus biofilms, through administration either as combination treatments or, more potently, as nitroxide functionalized ciprofloxacin derivatives. Although this study has focused on fluoroquinolones, a class of antibiotics widely used against many common infections, the methodology presented is easily adaptable to other antibiotic and antimicrobial classes, widely expanding the possible repertoire of antibiofilm agents that are so urgently needed. This work serves as an early preclinical evaluation of nitroxide functionalized antibiotics as new antimicrobials for the treatment of S. aureus biofilms and showcases a promising therapeutic strategy with broad-spectrum antibiofilm potential.

## MATERIALS AND METHODS

### Bacterial and human cell culture media and conditions.

Staphylococcus aureus strain ATCC 29213 was used in this study. Bacteria were grown routinely in lysogeny broth (LB) at 37°C, with shaking (200 rpm). MIC assays were conducted in Mueller-Hinton (MH) medium (Oxoid, Thermo Fisher, Australia), biofilms were grown in LB, and biofilm challenges (dispersal and antimicrobial susceptibility testing) were performed in MH medium or M9 minimal medium (pH 7.0) containing 90 mM Na_2_HPO_4_, 22 mM KH_2_PO_4_, 9 mM NaCl, 19 mM NH_4_Cl, 2 mM MgSO_4_, 100 μM CaCl_2_, and 22 mM glucose. Human T24 bladder epithelial cells (HTB-4, obtained from ATCC) were cultured in McCoy’s 5A modified medium (Thermo Fisher) supplemented with 10% heat-inactivated fetal bovine serum (Thermo Fisher). Confluent monolayers were formed by cell culture at 37°C in a humidified atmosphere with 5% CO_2_.

### Nitroxide and antibiotic stock solutions.

The nitroxides CTEMPO (Sigma, Australia) and CTMIO (48) were prepared in dimethyl sulfoxide (DMSO) at a concentration of 325 mM (stock solution). The antibiotic ciprofloxacin (Sigma) was prepared in aqueous hydrochloric acid (0.1 M) at a concentration of 60.35 mM (stock solution). Cipro-PROXYL, cipro-TEMPO, cipro-TMIO, fluoroquinolone-TEMPO, and fluoroquinolone-TEMPOMe were synthesized in house, utilizing previously established procedures (25, 26), and stock solutions were prepared in DMSO (8 mM). All stock solutions were stored in the absence of light at −20°C. Working solutions were prepared in either M9 minimal medium or MH medium and were used the same day.

### Nitroxide and antibiotic MIC and MBC assays.

The MICs for CTEMPO, CTMIO, ciprofloxacin, cipro-PROXYL, cipro-TEMPO, cipro-TMIO, fluoroquinolone-TEMPO, and fluoroquinolone-TEMPOMe were determined by the broth microdilution method, in accordance with Clinical and Laboratory Standards Institute (CLSI) guidelines (49). Specifically, in a 96-well plate, twelve 2-fold serial dilutions of each compound were prepared in MH medium at a final volume of 100 μl. A positive-control sample (bacteria only) and a negative-control sample (medium only) were also included. At the initial time of inoculation, each well was inoculated with 5 × 10^5^ CFU, which had been prepared from fresh overnight cultures. MICs were determined visually at the 18-h endpoint. The MIC was defined as the lowest concentration of a compound that prevented visible bacterial growth after 18 h of static incubation at 37°C. MBC values were determined by subculturing 20 μl from each well of the final MIC challenge plate (after 18 h of incubation) in 180 μl of fresh MH medium in a 96-well plate, followed by static incubation for 24 h at 37°C. MBCs were determined visually at the 24-h endpoint. The MBC was defined as the lowest concentration of agent required to eradicate ≤99.9% of the cells (as indicated by clear wells). CTEMPO and CTMIO were tested in the concentration range of 3,200 to 1.56 μM, ciprofloxacin in the concentration range of 160 to 0.048 μM, and cipro-PROXYL, cipro-TEMPO, cipro-TMIO, fluoroquinolone-TEMPO, and fluoroquinolone-TEMPOMe in the concentration range of 1,200 to 0.3 μM. Negative-control samples containing DMSO (vehicle control for CTEMPO, CTMIO, cipro-PROXYL, cipro-TEMPO, cipro-TMIO, fluoroquinolone-TEMPO, and fluoroquinolone-TEMPOMe) were prepared and serially diluted as described above. MIC and MBC testing was conducted in at least two independent experiments, each containing three culture replicates tested in duplicate technical repeats. The reported MIC and MBC values were determined as the lowest concentrations at which all replicates exhibited no visible growth. No variation between replicates was observed.

### Biofilm culture using the Calgary biofilm device.

Biofilms were grown and established in the Calgary biofilm device (CBD) (MBEC assay; Innovotech Inc., Canada), which was used unmodified. The device consists of a two-part reaction vessel; the top component contains 96 identical pegs protruding down from the lid, which fits into a standard, flat-bottom, 96-well plate (the bottom component). Biofilm cultures were performed as described previously (50). Briefly, overnight bacterial cultures in LB were diluted to 10^6^ CFU/ml in LB and used to inoculate the enclosed, flat-bottom, 96-well plate with ∼10^5^ bacterial cells (130 μl) in each well. The peg lid was inserted into the inoculated wells, and the complete CBD was incubated for 24 h in a shaking incubator at 150 rpm, at 37°C in 95% relative humidity.

### Nitroxide, antibiotic, cotreatment, and hybrid compound MBEC assays.

Biofilms were established as detailed above. For MBEC determinations, the CBD lid containing established biofilms was removed and rinsed for 10 s in phosphate-buffered saline (PBS) (96-well plate, with 200 μl in each well), to remove loosely adherent bacteria. The rinsed CBD lid was then transferred to a new flat-bottom, 96-well plate containing the specific treatment (challenge plate). For nitroxides alone, the challenge plate contained 2-fold serial dilutions with a concentration range of 1,200 to 9.4 μM for CTMIO or a concentration range of 2,048 to 16 μM for CTEMPO. For ciprofloxacin alone, the challenge plate contained 2-fold serial dilutions with a concentration range of 4,096 to 32 μM. For cotreatments (ciprofloxacin with CTEMPO or CTMIO), the challenge plate contained 2-fold serial dilutions of ciprofloxacin (concentration range of 4,096 to 32 μM) with CTMIO (tested only at 8 μM) or CTEMPO (at a consistent concentration in each well of 80, 40, 20, 10, 8, or 2.5 μM) in M9 minimal medium, in a total volume of 200 μl per well. For hybrid compounds, the challenge plate contained 2-fold serial dilutions with a concentration range of 1,024 to 8 μM for cipro-PROXYL, cipro-TEMPO, cipro-TMIO, fluoroquinolone-TEMPO, or fluoroquinolone-TEMPOMe. The complete CBD was then incubated for 24 h at 37°C in 95% relative humidity, at 120 rpm. The lid was removed from the challenge plate and rinsed twice for 30 s in PBS (96-well plate, with 200 μl in each well). The rinsed CBD lid, with attached pegs and treated biofilms, was transferred to a new 96-well plate containing fresh LB recovery medium. To facilitate the transfer of any remaining viable cells to the recovery medium, the device was sonicated for 30 min at <20°C. The peg lid was then discarded, the biofilm-recovered bacteria from each well of the recovery plate were serially diluted in PBS, and then triplicate 5-μl aliquots of each dilution were plated onto LB agar and incubated overnight at 37°C for viable CFU enumeration. MBEC values were determined as the lowest concentrations that resulted in CFU values that were ≤99.9% of the untreated control values. MBEC values were obtained from at least two independent experiments, each consisting of three biological replicates assessed in triplicate technical repeats.

### Nitroxide-mediated biofilm dispersal assays.

Biofilm dispersal assays were performed as described above for MBEC assays, with the following modifications and additional steps. The challenge plate contained 2-fold serial dilutions of CTEMPO (concentration range of 160 to 2.5 μM) or CTMIO (8 μM) with ciprofloxacin at a consistent concentration of 6 μM (included to eradicate dispersed cells) in M9 minimal medium, in a total volume of 200 μl per well; control wells contained only ciprofloxacin at 6 μM. After sonication, the peg lid was discarded, and the recovery plate containing biofilm-recovered bacteria was covered with a breathable sealing membrane (Breathe-Easy sealing membrane; Sigma) and incubated for 20 h at 37°C, with shaking, in a microtiter plate reader (BMG, Australia). Measurements of the optical density at 600 nm were obtained every 15 min over the 20-h period. Growth curves for each well containing recovered biofilm cells were then plotted, and a nonlinear regression function (solver) was applied to determine the lag time in each growth curve (lag time is directly proportional to the initial CFU count of each well, with wells initially containing low CFU counts producing longer lag times than wells with higher initial CFU counts) (51, 52). Lag time was determined for each replicate for each condition tested, and group medians were compared by a Kruskal-Wallis test (in GraphPad Prism 7).

### CLSM of S. aureus biofilms.

Biofilms were grown on the CBD as detailed above. Established biofilms were treated with fluoroquinolone-TEMPO or fluoroquinolone-TEMPOMe (150 μM) in M9 medium, incubated at 37°C for 2 h, rinsed in PBS for 5 s, and stained with the LIVE/DEAD *Bac*Light bacterial viability kit (product no. L7007; Life Technologies, Australia), according to the manufacturer’s protocol. Treated and stained biofilms were mounted using ProLong Diamond antifade mountant (Life Technologies) and were immediately analyzed by CLSM. CLSM was conducted with a Zeiss 780 NLO point scanning confocal microscope equipped with a Mai-Tai DeepSee multiphoton laser (tunable between 690 and 1,040 nm). The Mai-Tai laser was set at 720 nm (a wavelength that did not excite SYTO9 or propidium iodide [PI]) for fluoroquinolone-TEMPO and fluoroquinolone-TEMPOMe, the 488-nm laser was used for SYTO9, and the 561-nm laser was used for PI. CLSM z-slices were obtained every 0.5 μm throughout the thickness of each biofilm (approximately 10 to 20 μm). Image stacks were analyzed using the instrument software (Zen 2.3). All imaging experiments utilized a 100× oil immersion objective.

### LDH release cytotoxicity assay.

The cytotoxicity of fluoroquinolone-TEMPO and CTEMPO against human T24 urinary bladder epithelial cells was examined utilizing the Pierce LDH cytotoxicity assay kit (Life Technologies), according to the manufacturer’s instructions. Briefly, triplicate confluent T24 cell monolayers were treated with fluoroquinolone-TEMPO or CTEMPO (concentrations between 720 and 20 μM) for 24 h at 37°C, in a humidified atmosphere of 5% CO_2_. Cells treated with DMSO in PBS (final concentration of 4.5% DMSO) or sterile water served as negative-control samples, and cells treated with 10× lysis buffer (maximum LDH release) served as positive-control samples. After 24 h of incubation, 50 μl of the supernatant was transferred to a new 96-well plate, mixed with 50 μl of the reaction mixture (LDH assay kit), and incubated at room temperature (protected from light) for 30 min before the stop solution (50 μl) was added. The plate was then centrifuged at 1,000 × *g* for 5 min to remove air bubbles, and the absorbance at 490 and 680 nm was measured in a SPECTROstar plate reader (BMG).
